# Dietary of different forms of *Humulus scandens* on growth performance and intestinal bacterial communities in piglets

**DOI:** 10.1093/tas/txad139

**Published:** 2023-12-23

**Authors:** Lihong Hao, Cheng Wang, Huaizhong Wang, Meng Zhou, Yong Wang, Hongmei Hu

**Affiliations:** Key Laboratory of Livestock and Poultry Multi-omics of MARA, Institute of Animal Science and Veterinary Medicine, Shandong Academy of Agricultural Sciences, Jinan 250000, China; Shandong Key Laboratory of Animal Disease Control and Breeding, Institute of Animal Science and Veterinary Medicine, Shandong Academy of Agricultural Sciences, Jinan 250000, China; Key Laboratory of Livestock and Poultry Multi-omics of MARA, Institute of Animal Science and Veterinary Medicine, Shandong Academy of Agricultural Sciences, Jinan 250000, China; Shandong Key Laboratory of Animal Disease Control and Breeding, Institute of Animal Science and Veterinary Medicine, Shandong Academy of Agricultural Sciences, Jinan 250000, China; Key Laboratory of Livestock and Poultry Multi-omics of MARA, Institute of Animal Science and Veterinary Medicine, Shandong Academy of Agricultural Sciences, Jinan 250000, China; Shandong Key Laboratory of Animal Disease Control and Breeding, Institute of Animal Science and Veterinary Medicine, Shandong Academy of Agricultural Sciences, Jinan 250000, China; Business Environment Promotion Department, Jinan Enterprise Service Center, Central, Jinan 250000, China; Environmental Protection Equipment Department, Jinan Department of Husbandry Extension, Changqing, Jinan 250000, China; Key Laboratory of Livestock and Poultry Multi-omics of MARA, Institute of Animal Science and Veterinary Medicine, Shandong Academy of Agricultural Sciences, Jinan 250000, China; Shandong Key Laboratory of Animal Disease Control and Breeding, Institute of Animal Science and Veterinary Medicine, Shandong Academy of Agricultural Sciences, Jinan 250000, China

**Keywords:** bacterial communities, *Humulus scandens*, nutrient digestibility, piglet, serum immunity

## Abstract

The present study was aimed at elucidating the effects of feeding different forms of *Humulus scandens* (Hu) on performance and bacterial communities in piglets. A total of 160 piglets were divided into four groups: (1) a control (CG, basal diet); (2) a basal diet with Hu pulp (HS), basal diet + Hu pulp; (3) a basal diet with Hu juice (HSJ), basal diet + Hu juice; and (4) a basal diet with Hu residue (HSR), basal diet + Hu residue. Results showed that HS, HSJ, and HSR supplementation led to rich average daily gain (**ADG**) and poor feed conversion ratio (**FCR**) during 28 to 70 d of age, increased 120 d body weight (**BW**), average daily feed intake (**ADFI**) and ADG and decreased FCR during 71 to 120 d of age. Three experiment groups presented greater (*P* < 0.05) IgA, IgG, and IgM and lower (*P* < 0.05) glucose, and blood urea nitrogen. The content of diamine oxidase significantly decreased (*P* < 0.05) in HS group. The crude  protein and crude fiber digestibility were improved (*P* < 0.05) in HS group and the Ca digestibility was increased (*P* < 0.05) in HS and HSJ groups. HSR supplementation improved the abundance of *Firmicutes* and decreased the abundance of *Bacteroidetes*. Hu supplementation with different forms increased the proportion of *Lactobacillus* in cecum content. These results indicated that supplemental feeding of Hu with different forms improved serum immunity, nutrient digestibility, and bacterial communities in piglets, promoting growth and development, which may be regarded as a reference for developing novel feed resources for piglets.

## Teaser

This study revealed that supplemental feeding of piglets with HS, HSJ, and HSR improved serum immunity, nutrient digestibility, and bacterial communities, leading to enhanced growth performance. These findings may be regarded as a reference for developing novel feed resources for piglets.

## Introduction

*Humulus scandens* (**Hu**), a dioecious perennial twinning or creeping vine, pertains to the family of *Cannabaceae* and usually grows as a weed in China and Korea, where it is also referred to as *Humulus japonicus* (Sung et al., 2015). Depending on wind pollination, Hu exerts the flowering stage from August to October and grows rapidly as a vine, subsequently forming dense stands and replacing native vegetation by competing for basic resources (Lim et al., 2016). In traditional medicine, Hu is widely used to conquer diarrhea, tuberculosis, pneumonia, dysentery, pulmonary, hypertension, leprosy, and promote blood circulation (Naya et al., 1970; Kumar et al., 2007; Sung et al., 2015). While the pharmacological effects of Hu may be attributed to its extracts possessing potent anti-inflammatory (Wang et al., 2021), antimicrobial (Zhang et al., 2014), antimycobacterial (Hong et al., 2014), antiatherogenic (Lim et al., 2016) and anticancer (Lee et al., 2012) functions. Additionally, previous studies reported the positive effects of Hu extracts on Alzheimer’s disease protection and active oxygen molecule scavenging (Kang et al., 2022; Wang et al., 2022). The bioactive components of Hu are the foundation for its biological functions. Flavone glycosides, terpenes, alkaloids, lupulones, phenolics, isoquinoline, flavonoids, and polysaccharides were observed in the extracts of Hu (Yan et al., 2011; Sung et al., 2015; Wang et al., 2015; Hao et al., 2022). Furthermore, Hu extracts have also been reported to contain amino acids, vitamins, and trace minerals (Feng and Qi, 2009; Zhang et al., 2014, 2021).

Researches have shown that herbs or their extracts contributed to improving the production performance of animals, which might produce effects comparable to antibiotics (Oso et al., 2019; Dang et al., 2021). Hu is not excepted. It is reported that dietary Hu ethanol extract supplementation improved the milk yield in dairy cows (Li, 2012). A study by Guo et al. (2008) has revealed that feeding fattening sheep with 2.5 kg fresh Hu increased the feed conversion rate and reduced feed costs. Chen et al. (2001) demonstrated that dietary supplementation of Hu powder in growing-finishing pigs resulted in the increase of average daily gain (**ADG**). Furthermore, Hu water extract has been reported to regulate the lipid metabolism by decreasing serum total cholesterol, low-density lipoprotein, and triglyceride and increasing high-density lipoprotein in broiler chicks (Zhao et al., 2017). Liu et al. (2008) suggested that dietary supplementation of Hu ethanol extract contributed to improving the protein and energy digestibility and growth performance in grass carp. Our previous study demonstrated that Hu after crushing was proportionally added to diets, which possessed positive effects on nutrient digestibility and gut microbiota modulation, thus improving growth performance in piglets (Hao et al., 2022). However, studies on the effects of Hu with different forms on growth performance and bacterial communities in piglets are very scarce.

Here, the main objective of this study was to evaluate the effects of Hu with different forms on growth performance, nutrient digestibility, serum immunity, and bacterial communities of piglets, which contributed to further understanding the application of Hu on piglets for the development of new feed resources for promoting piglet growth.

## Materials and Methods

The experimental protocol was performed strictly according to guidelines (IACC20060101, January 1, 2006) of the Institutional Animal Care and Use Committee of the Institute of Animal Science and Veterinary Medicine, Shandong Academy of Agricultural Sciences.

### Preparation of the Hu Diets

The fresh whole plant Hu was harvested and crushed to be slurry with a pulper, which was Hu pulp. Then a juicer was used to separate the juice and residue by pressing, which were Hu juice and Hu residue. The chemical analysis of Hu has been reported in our previous study (Hao et al., 2022).

### Experimental Design and Diets

The basal diet (Table 1) was formulated to meet the NRC (2012) nutrient requirements. The Hu pulp, Hu juice, and Hu residue were then proportionally added to diets according to the experimental design below. One hundred and sixty piglets (Duroc × Landrace × Large White), 21 d old, with an initial body weight (**BW**) of 6.62 ± 0.50 kg, were randomly allocated into 4 groups based on BW and sex (50% males and 50% females, 5 replicates per group and 8 piglets per replicate) after one week of pre-feeding. Dietary treatments were: (1) CG, basal diet; (2) HS, basal diet + Hu pulp; (3) HSJ, basal diet + Hu juice; (4) HSR, basal diet + Hu residue. Experiment 1. Each pig in HS groups was fed the basal diet supplemented with 30, 40, and 50 g Hu pulp from 28 to 37, 38 to 54, and 55 to 70 d of age, respectively. Each pig in HSJ groups was fed the basal diet supplemented with all the juice separated from 30, 40, and 50 g Hu after pressing during 28 to 37, 38 to 54, and 55 to 70 d of age, respectively. Each pig in HSR groups was fed the basal diet supplemented with all the residue separated from 30, 40, and 50 g Hu after pressing during 28 to 37, 38 to 54, and 55 to 70 d of age, respectively. Piglets in the control group were fed the Dabei Nong’s full-price feed of the conservation period during 28 to 70 d of age. Experiment 2: After 28 Experiment 70 d of age, all pigs according to the original group were fed the Dabei Nong’s full-price feed of the growth period and the experiment ended at the age of 120 d. The pigs had provided ad libitum access to feed and water throughout the experiment.

**Table 1.** Composition and nutritional levels of basal diet (air-dried basis)

Ingredients	Formula		Nutrients^1^	Contents	
	Days 28 to 70	Days 71 to 120		Days 28 to 70	Days 71 to 120
Corn	65.00	65.00	DE, MJ/kg	13.38	13.22
Soybean meal	31.00	27.00	CP, %	18.46	17.36
Wheat bran	0.40	4.75	CF, %	2.89	2.96
Dicalcium phosphate	0.95	0.55	Ca, %	0.75	0.62
Sodium chloride	0.25	0.30	P, %	0.59	0.52
Limestone power	0.90	0.90	Lys, %	0.98	0.89
Premix^2^	1.50	1.50	Met + Cys, %	0.61	0.52
Total	100.00	100.00	Thr, %	0.63	0.49

DE, digestible energy; CP, crude protein; CF, crude fiber; Ca, calcium; P, phosphorus, Lys, lysine, Met, Methionine, Cys, cysteine, Thr, Threonine.

^1^DE was a calculated value, while the others were measured values.

^2^The premix provided per kilogram of diets: vitamin A, 6,000 IU; vitamin D_3_, 100 IU; vitamin E, 26 IU; vitamin K_3_, 2 mg; vitamin B_1_, 8.8 mg; vitamin B_2_, 2.4 mg; vitamin B_6_, 7 mg; niacin, 14 mg; folic acid, 0.68 mg; vitamin H, 0.2 mg; Fe, 60 mg; Cu, 30 mg; Mn, 30 mg; Zn, 85 mg.

### Growth Performance

Piglets were weighed on an empty stomach head by head at the beginning, 70 d of age, and end of the experiment for determining the ADG. The feed consumption of each replicate pen was recorded to calculate the average daily feed intake (**ADFI**) and feed conversion ratio (**FCR**).

### Sample Collection

At 70 d of age, pigs were disinfected with alcohol on the neck after fasted for 12 h,s and 10 mL blood from each pig was extracted in uncoated vacuum blood collection tubes. Subsequently, the serum was separated and stored at −80 °C after centrifugation at 3500 rpm for 15 min for serum biochemical and immunoglobulins indices analysis. The fecal samples were collected by massaging rectum daily from each pig for three days before the end of the experiment and mixed with 10% sulfuric acid for minimizing nitrogen loss before nutrient apparent digestibility determination. Fresh pig feces were gathered in 50 mL centrifuge tubes with disposable sterile gloves followed by rapidly freezing in liquid nitrogen and repositing at −80 °C to extract microbial DNA and analyze the characterization of fecal microbiota.

### Serum Biochemical and Immune Indices

The commercial assay kits (Nanjing Jiancheng Biological Product Co. Ltd., China) were applied to quantify the IgA, IgG, IgM, glucose (**GLU**), diamine oxidase (**DAO**), globulin (**GLO**), total protein (**TP**), blood urea nitrogen (**BUN**), Ca and P levels according to the manufacturers’ introductions.

### Nutrient apparent total tract digestibility analysis

Chromic oxide (0.2%) was used as an inert indicator for apparent total tract digestibility (**ATTD**) analysis. The fecal samples were dried in an oven at 70 °C for 72 h, crushed, and passed through a 1 mm screen. The UV absorption spectrophotometry (UV-1201; Shimadzu, Kyoto, Japan) was used to determine the chromium levels. The ATTD was measured according to the method of AOAC (2012). Parr 6100 oxygen bomb calorimeter (Parr Instrument Co., Moline, IL, USA) was applied to quantify the gross energy through the heat of combustion of samples analysis. The procedures used for ATTD calculation were conducted with indirect methods according to the methods described by Fenton and Fenton (1979).

### Microbial Analysis of Cecum Contents

The stool DNA kit (Omega Bio-tek, Norcross, GA, USA) was applied to extract total DNA from fecal samples according to the manufacturer’s protocols. The procedures used for DNA concentration and purity were conducted by NanoDrop 2000 (Thermo Scientific, Wilmington, USA), followed by 1% agarose gel electrophoresis examining DNA quality. The V3 to V4 variable region of 16S rRNA was amplified with 338F (5ʹ-ACTCCTACGGGAGGCAGGAG-3ʹ) and 806R (5ʹ-GGACTACHVGGGTWTCTAAT-3ʹ) as primers using the extracted total DNA as template and subsequently purified by QIAquick gel extraction kit (QIAGEN, Germany). The pure products were quantified with Quant-iT PicoGreen dsDNA assay kit (Life Technologies, Carlsbad, USA). The 16S rRNA libraries were built according to the method presented by Mao et al. (2016). After MisSeq genome sequencing, QIIME package (V1.7.0, http://qiime.org/scripts/split_libraries_fastq.html) was utilized to analyze the data. The main differentially abundant genera were screened according to the method of LEfSe (https://huttenhower.sph.harvard.edu/lefse/). The metabolic genes were predicted from 16S rRNA data using PICRUSt (https://github.com/picrust/picrust2/wiki). Then PICRUSt and KEGG were responsible for distributing predicted functional genes into metabolic pathways.

### Statistical Analysis

Statistical analysis was performed with SPSS software (SAS Inc., Chicago, IL). One-way ANOVA and Tukey’s multiple comparison analysis were applied to determine the statistical significance. Data were represented by means of SEM. The statistical significance level was considered significant at *P* < 0.05.

## Results

### Growth Performance

The effects of different forms of Hu on the growth performance of piglets aged 28 to 70 d are shown in Table 2. Compared with the control group, the ADG in HS and HSR groups were significantly increased (*P* < 0.05) by 11.04% and 9.42%, respectively, whereas that in HSJ group was not significant (*P* > 0.05). The ADG in HS group was greater than in HSJ group (*P* < 0.05). Moreover, no significant differences were observed between HS and HSR, HSJ, and HSR groups. Hu supplementation in HS, HSJ, and HSR groups significantly decreased (*P* < 0.05) the FCR by 12.35%, 14.12%, and 8.24%, respectively, in comparison to control group. The FCR in HSR group was greater than HSJ group (*P* < 0.05). No significant differences were found in HS and HSJ, HS and HSR groups (*P* > 0.05).

**Table 2.** Effects of supplementation with different forms of Hu on the growth performance of piglets aged 28 to 70 d

Item	CG	HS	HSJ	HSR	SEM	*P* value
Live weight						
Initial BW, kg	6.61	6.77	6.64	6.47	0.23	0.979
70 d BW, kg	26.46	28.81	27.11	28.19	0.66	0.664
ADFI, g/d	674.87	656.82	597.72	677.68	18.18	0.632
ADG, g/d	397.00^c^	440.82^a^	409.40^bc^	434.41^ab^	8.38	0.003
FCR	1.70^a^	1.49^bc^	1.46^c^	1.56^b^	0.02	<0.001

^a,b^Means with different superscripts in the same row within the trial differ (*P* < 0.05). Hu, *Humulus scandens*. CG, basal diet; HS, basal diet + Hu pulp; HSJ, basal diet + Hu juice; HSR, basal diet + Hu residue. BW, body weight; ADFI, average daily feed intake; ADG, average daily gain; FCR, feed conversion ratio. SEM, standard error of the mean.

The effects of different forms of Hu on growth performance of piglets aged 71 to 120 d are shown in Table 3. Compared with the control group, the 120 d BW, ADFI, and ADG in HS, HSJ, and HSR groups were significantly increased (*P* < 0.05), and no significant differences were observed in 120 d BW, ADFI, and ADG among the three experiment groups (*P* > 0.05). The FCR in HS and HSJ groups were significantly decreased (*P* < 0.05) by 13.99% and 11.26%, respectively, in comparison to control group, whereas that in HSR group was not significant (*P* > 0.05). Moreover, HSR group possessed greater (*P* < 0.05) FCR than HS and HSJ groups. No significant differences were found in CG and HSR, HS and HSJ groups (*P* > 0.05).

**Table 3.** Effects of supplementation with different forms of Hu on the growth performance of piglets aged 71 to 120 d

Item	CG	HS	HSJ	HSR	SEM	*P* value
Live weight						
70 d BW, kg	26.46	28.81	27.11	28.19	0.66	0.664
120 d BW, kg	41.59^b^	54.14^a^	52.10^a^	51.48^a^	1.83	0.024
ADFI, g/d	884.25^b^	1282.04^a^	1302.81^a^	1341.07^a^	56.93	0.002
ADG, g/d	302.62^b^	506.63^a^	499.82^a^	465.81^a^	24.95	0.003
FCR	2.93^a^	2.52^b^	2.60^b^	2.88^a^	0.05	<0.001

^a,b^Means with different superscripts in the same row within the trial differ (*P* < 0.05). Hu, *Humulus scandens*. CG, basal diet; HS, basal diet + Hu pulp; HSJ, basal diet + Hu juice; HSR, basal diet + Hu residue. BW, body weight; ADFI, average daily feed intake; ADG, average daily gain; FCR, feed conversion ratio. SEM, standard error of the mean.

### Serum Biochemical and Immune Function Indices

The effects of different forms of Hu on serum biochemical and immune indexes of piglets aged 70 d are shown in Table 4. Compared with the control group, the GLU in HS, HSJ, and HSR groups were significantly decreased (*P* < 0.05) by 17.71%, 15.20%, and 10.19%, respectively, and no significant differences were observed among the three groups (*P* > 0.05). The level of DAO significantly decreased (*P* < 0.05) in HS group compared to the other three groups, and the difference among the three groups was not significant (*P* > 0.05). Furthermore, Hu supplementation in HS, HSJ, and HSR groups significantly decreased (*P* < 0.05) the BUN level by 10.29%, 9.65%, and 9.32%, whereas there was no significant difference among the three groups (*P* > 0.05). As expected, the amounts of IgA, IgG, and IgM in serum were greater (*P* < 0.05) in HS, HSJ, and HSR groups in comparison to the control group, and no significant differences were observed in IgA, IgG, and IgM among the three groups (*P* > 0.05).

**Table 4.** Effects of supplementation with different forms of Hu on serum biochemical and immune indexes of piglets aged 70 d

Item	CG	HS	HSJ	HSR	SEM	*P* value
GLU, mmol/L	6.38^a^	5.25^b^	5.41^b^	5.73^b^	0.09	<0.001
TP, g/L	60.80	60.38	59.47	58.16	0.44	0.155
GLO, g/L	33.80	34.46	33.77	33.86	0.42	0.931
DAO, U/L	0.12^a^	0.09^b^	0.12^a^	0.12^a^	0.00	<0.001
BUN, mmol/L	3.11^a^	2.79^b^	2.81^b^	2.82^b^	0.04	0.001
IgA, mg/L	12.80^b^	17.50^a^	18.12^a^	16.78^a^	0.58	0.003
IgG, g/L	1.61^b^	1.97^a^	2.15^a^	2.02^a^	0.04	<0.001
IgM, g/L	0.17^b^	0.24^a^	0.26^a^	0.24^a^	0.01	<0.001
Ca, mmol/L	2.76	2.53	2.51	2.55	0.04	0.168
P, mmol/L	3.48	3.09	3.25	3.10	0.06	0.078

Values are means of 12 replicates per treatment. ^a,b^Means with different superscripts in the same row within the trial differ (*P* < 0.05). Hu, *Humulus scandens*. CG, basal diet; HS, basal diet + Hu pulp; HSJ, basal diet + Hu juice; HSR, basal diet + Hu residue. GLU, glucose; TP, total protein; GLO, globulin; DAO, diamine oxidase; BUN, blood urea nitrogen; Ca, calcium; P, phosphorus. SEM, standard error of the mean.

### Nutrient Apparent Digestibility

The results of nutrient apparent digestibility analysis in piglets aged 70 d during the metabolism trial are shown in Table 5. Compared with the control group, the total protein (**CP**) and crude fiber (**CF**) digestibility in HS group were significantly increased (*P* < 0.05) by 7.22% and 8.27%, respectively, and those in HSJ and HSR groups were not significant (*P* > 0.05). In comparison to HS group, the CP and CF level were significantly decreased (*P* < 0.05) in HSR group, whereas there were no significant changes in HSJ group (*P* > 0.05). The digestibility of Ca was significantly increased (*P *< 0.05) in HS and HSJ groups compared to the control group, and that in HSR group was not significant (*P* > 0.05). Moreover, no significant differences were observed in Ca digestibility among HS, HSJ, and HSR groups (*P* > 0.05). The result also suggested that different forms of Hu supplementation in HS, HSJ, and HSR groups tended to increase the digestibility of ether extract (**EE**) and phosphorus (P).

**Table 5.** Effects of supplementation with different forms of Hu on the nutrient apparent digestibility of piglets aged 70 d

Item	CG	HS	HSJ	HSR	SEM	*P* value
CP digestibility, %	75.15^b^	80.58^a^	78.11^ab^	76.67^b^	0.57	0.004
CF digestibility, %	46.32^b^	50.15^a^	47.21^ab^	45.28^b^	0.51	0.003
EE digestibility, %	45.45	48.85	47.55	45.74	0.50	0.055
Ca digestibility, %	65.20^b^	70.35^a^	69.17^a^	67.09^ab^	0.56	0.003
P digestibility, %	59.04	65.35	63.18	60.46	0.97	0.092

Values are means of 12 replicates per treatment. ^a,b,c^Means with different superscripts in the same row within the trial differ (*P* < 0.05). Hu, *Humulus scandens*. CG, basal diet; HS, basal diet + Hu pulp; HSJ, basal diet + Hu juice; HSR, basal diet + Hu residue. CP, crude protein; CF, crude fibre; EE, ether extract. Ca, calcium; P, phosphorus. SEM, standard error of the mean.

### Bacterial Diversity and Community Structure

**Figure 1.**
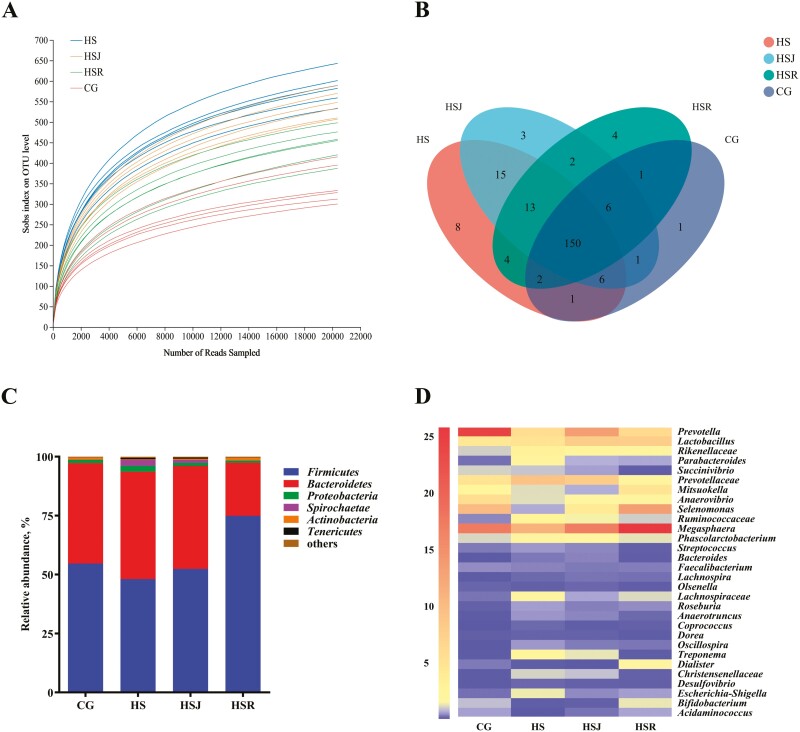
Bacterial diversity and community structure during feeding phase (*n* = 6). (A) Observed operational taxonomic unit line chart. (B) Venn diagram representing the common and unique OTUs found in each treatment. (C) Phylum-level compositions of the bacterial community of piglets. (D) Genus-level compositions of the bacterial community of piglets. CG, basal diet; HS, basal diet + Hu pulp; HSJ, basal diet + Hu juice; HSR, basal diet + Hu residue.

The bacterial diversity and community structure in the cecum of piglets after Hu administration were analyzed by 16S rRNA sequencing. As shown in Table 6, compared with the control group, the Sobs and Chao index were significantly increased (*P* < 0.05) in HS, HSJ, and HSR groups. Moreover, HS and HSJ groups possessed greater (*P* < 0.05) Sobs and Chao index than HSR group, whereas no significant differences were observed between HS and HSJ groups (*P* > 0.05). The Shannon index between CG and HSR groups was similar and lower (*P* < 0.05) than in the HS and HSJ groups and no significant differences were observed between HS and HSJ groups (*P *> 0.05). The Ace index was significantly increased (*P *< 0.05) in all treatment groups in comparison to control group, and HS group possessed greater (*P* < 0.05) Ace index than HSR group. Moreover, there were no significant differences in HS and HSJ, HSJ, and HSR groups (*P* > 0.05). Compared with the control group, HS group showed a significant decrease (*P* < 0.05) in Simpson index, while HSJ and HSR groups showed no significant changes (*P* > 0.05). Moreover, the Simpson index between HS and HSJ groups was similar and lower than in HSR group (*P* < 0.05). The result of the coverage index implied that the abundant operational taxonomic unit (OTU) coverage and sufficient sequencing depth could assess the bacterial community in the cecum of piglets during the feeding phase. Figure 1A presented that the number of OTUs was increased in diets supplemented with HS, HSJ, and HSR. The common and unique OTUs among groups are shown in Figure 1B. There were one hundred and fifty OTUs as the core genera in four groups.

**Table 6.** Effects of supplementation with different forms of Hu on alpha diversity indices of cecum bacteria in piglets aged 70 d

Item	CG	HS	HSJ	HSR	SEM	*P*-value
Sobs index	347.17^c^	584.50^a^	543.00^a^	449.00^b^	20.51	<0.001
Chao index	439.66^c^	679.01^a^	661.11^a^	570.37^b^	22.40	<0.001
Ace index	434.25^c^	674.09^a^	652.69^ab^	574.81^b^	22.02	<0.001
Shannon index	3.68^b^	4.57^a^	4.35^a^	3.80^b^	0.09	<0.001
Simpson index	0.06^ab^	0.03^c^	0.04^bc^	0.07^a^	0.00	<0.001
Coverage index	99.56	99.41	99.37	99.43	0.02	0.061

Values are means of 6 replicates per treatment. ^a,b,c^Means with different superscripts in the same row within the trial differ (*P* < 0.05). Hu, *Humulus scandens*. CG, basal diet; HS, basal diet + Hu pulp; HSJ, basal diet + Hu juice; HSR, basal diet + Hu residue. SEM, standard error of the mean.

For further analysis of bacterial community, the taxon abundances among groups were compared by 16S rRNA libraries. Figure 1C displays the relative abundances at the phylum level in the cecum of piglets. In the present study, each group possessed above six bacterial phyla, accompanied by *Firmicutes*, *Proteobacteria*, and *Bacteroidetes* with the proportion of over 96%. Compared with the control group, the abundances of *Firmicutes* were lower in HS and HSJ groups (54.65 vs. 48.10%, 54.65 vs. 52.38%) and greater in HSR group (54.65 vs. 74.89%). On the contrary, HS and HSJ groups presented an increase trend of *Bacteroidetes*, while that of HSR group showed a decrease. Furthermore, dietary different forms of Hu increased the abundance of *Tenericutes*, whose proportion was 0.08%, 0.59%, 0.48%, and 0.16%, respectively, in four groups. The lowest abundances of *Proteobacteria*, *Spirochaetae*, and *Actinobacteria* were observed in HSR, GC, and HS groups, respectively (Supplementary Table S1). Figure 1D presents the relative abundances of different bacterial genera within different communities in the cecum of piglets. The dominant genera in control group were *Prevotella* (24.31%) followed by *Megasphaera* (17.38%) and *Selenomonas* (10.25%) while those in treatment groups were *Megasphaera* (18.00%), *Prevotella* (8.59%), and *Lactobacillus* (6.95%). As expected, Hu supplementation with different forms increased the abundance of *Lactobacillus*, whose proportion was 4.51%, 5.04%, 8.02%, and 7.81%, respectively, in four groups (Supplementary Table S2).

### Bacterial Metabolic Functions Prediction

**Figure 2.**
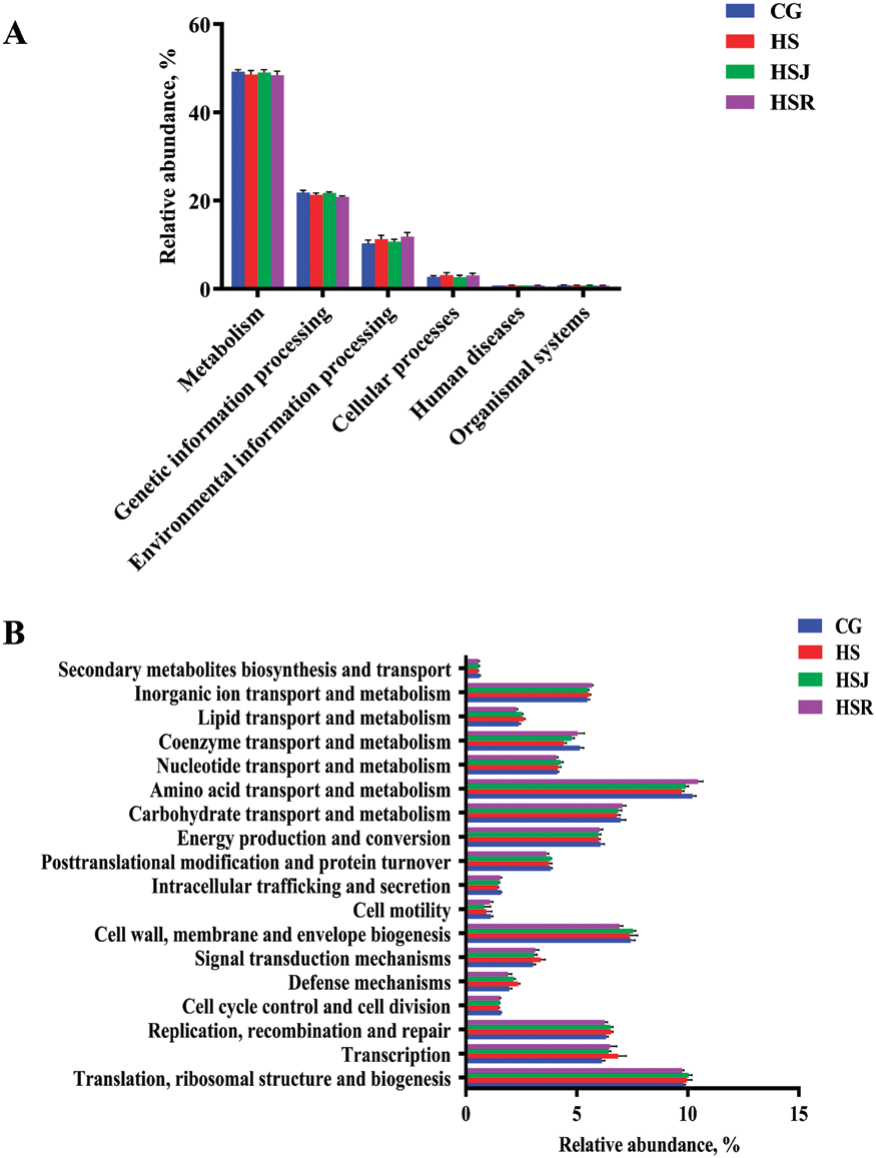
Bacterial metabolic functions in the cecum of piglets during feeding phase analyzed by PICRUSt (*n* = 6). (A) Level 1 metabolic pathways. (B) Level 2 KEGG ortholog functional predictions. CG, basal diet; HS, basal diet + Hu pulp; HSJ, basal diet + Hu juice; HSR, basal diet + Hu residue.

Figure 2 displays the bacterial metabolic functions in the cecum of piglets. Among six metabolic functions, the predicted protein sequences in four groups ranged from 49.16% to 0.74% (Figure 2A), accompanied by metabolism, environmental information processing and genetic information processing possessing over 81% (Supplementary Table S3). As shown in Figure 2B, amino acid transport and metabolism, translation, ribosomal structure and biogenesis and cell wall, membrane, and envelope biogenesis were the most abundant pathways during the feeding period. Furthermore, compared with the control group, HS and HSJ groups possessed greater (*P* < 0.05) replication, recombination and repair, defense mechanisms and lipid transport and metabolism and lower (*P* < 0.05) amino acid transport and metabolism and coenzyme transport and metabolism. The sequences related to transcription and signal transduction mechanisms were significantly increased (*P* < 0.05) in HS group when compared with the control group. HSR group possessed greater (*P* < 0.05) inorganic ion transport and metabolism and lower (*P *< 0.05) cell wall, membrane and envelope biogenesis, posttranslational modification and protein turnover and lipid transport and metabolism than control group (Supplementary Table S4).

## Discussion

For a long time, antibiotics as a feed additive possessed vital effects in addressing various issues in livestock husbandry, such as bacterial infections, weaning diarrhea and so forth. However, the European Commission gradually prohibited the use of antibiotics in animal feed after perceiving their dangers as early as 1997. Moreover, antibiotics as a growth promoter were completely prohibited in livestock husbandry until 2006 (Anadón, 2006). Animal nutritionists were subsequently devoted to developing antibiotic substitutes and herbs as long-term drug sources for humans have always been a priority (Gong et al., 2014). Previous researches indicated that some herbs have been shown to replace antibiotics for livestock, such as *Emblica officinalis*, *Aloe vera*, *Achyranthes Japonica Nakai* (Patel et al., 2016; Shokri et al., 2017; Dang et al., 2020). Hence, the present study was carried out to investigate the application of Hu, as herbs, with different forms on nutrient digestibility, serum immunity, bacterial community, and growth performance of piglets.

The use of herb feed additives was shown to reduce the cost of pig breeding and improve meat quality, which would contribute to better absorption ability of the intestinal tract and yield a better growth performance (Lin et al., 2020). Consistent with the previous study, our study revealed that HS, HSJ, and HSR supplementation led to rich ADG and poor FCR from 28 to 70 d of age, increased the 120 d BW, ADFI, and ADG and decreased FCR from 71 to 120 d of age. Moreover, the ADFI was decreased in HS and HSJ administration groups at 28 to 70 d of age in the present study. This is likely due to the presence of flavonoids which was reported in our previous study (Hao et al., 2022) and could decrease the feed palatability and intake owing to its astringency (Jansman, 1993). Furthermore, polyphenols, as another bioactive constituent from Hu (Zhang et al., 2021), possessed the effects of improving the feed conversion in pigs by manipulating gut microflora (Fiesel et al., 2014). Hence, the lower FCR rather than greater ADFI could be attributed to polyphenols, which might lead to an increase in dietary energy utilization through modulating the intestinal flora (Jami et al., 2012).

The decomposing metabolites produced during the growth and development of animals will eventually enter the blood, thus the serum biochemical indicators could reflect the health and metabolism status of animals (Lan et al., 2016). Serum GLU reflects the status of body glucose metabolism. In the present study, the serum GLU levels were decreased in Hu supplementation groups, which might be due to the inclusion of Hu in piglet diets lowering the absorption of carbohydrates in intestine. Jiao et al. (2015) reported that serum DAO concentration increasing indicated intestinal barrier damage. Lower DAO levels were observed in HS administration groups suggested that HS possessed the ability to decrease intestinal injury. As safeguards against pathogenic viruses and microbes, IgA, IgG, and IgM are critical ingredients accounting for mammalian humoral immunity (Ahmed et al., 2016). Katamaya et al. (2011) and Zhao et al. (2013) elicited that licorice root and *Ginkgo biloba* L. leaf as herbs contributed to improving the immune level of pigs and laying hens, respectively. Consistent with the previous researches, the present study noted that IgA, IgG, and IgM levels were greater in the administration groups than control groups, revealing that the immune levels were improved after Hu supplementation and the presence of flavonoids and polysaccharide might account for the results (Ren et al., 2021; Hoskin and Coombs, 2022). Serum BUN as the indicator of protein metabolism is the protein degradation product in vivo (Lin et al., 2020). In our study, supplemental feeding with Hu decreased the serum BUN levels, implying that more proteins were synthesized after Hu administration.

The previous study indicated that there existed an improvement of nutrients digestibility in finishing pigs fed with *Achyranthes japonica* extracts supplemented diet (Liu and Kim, 2023). In the same line, Guo et al. (2008) fed sheep with a fresh HS-containing diet and found that its supplementation possessed positive effects on the digestibility of EE and CF. Consistent with the previous findings, the present study showed that supplementing HS to the diet of piglets significantly increased CP and CF digestibility. Moreover, the digestibility of Ca was greater in HS and HSJ groups than control groups. The result of the study may be attributed to the accumulation of polyphenols in Hu according to a report from Sarker et al. (2010) who found that supplemental feeding with plant polyphenols significantly improved the dietary nutrient utilization of growing fattening pigs. Furthermore, previous research indicated that the digestion and absorption capacity of the small intestine in pigs was improved when supplemented with herb feed additives in the diet (Lin et al., 2020), which may contribute to better digestion and absorption of nutrients and partly explain the promotion of growth performance in the study.

The microbial community greatly affects the growth, development, and health condition of animals, especially the digestion and absorption of nutrients, as well as immune and physiological modulation (Ji et al., 2018). Moreover, Clarke et al. (2014) found that intestinal microbial diversity exerted vital roles in describing the health and metabolic capacity. The present study showed that the Sobs, Chao, Ace, and Shannon index were greater in the administration groups than control groups, revealing that Hu supplementation improved the richness and evenness of gut microbial community. In addition, the number of OTUs was increased in diets supplemented with Hu, and a total of one hundred and fifty OTUs were shared by the four groups, suggesting there existed strong core genera among these groups.

The influence of Hu administration on the composition of bacterial community of piglets was investigated through 16S rRNA high-throughput sequencing. *Firmicutes* and *Bacteroidetes* as two main communities accounted for regulating the homeostasis of host energy metabolism (Musso et al., 2010). Consistent with previous findings, our study revealed that the most abundant phyla were *Firmicutes* and *Bacteroidetes* in the cecum of piglets among the four groups. Moreover, the raise of *Firmicutes* accompanied by the decrease of *Bacteroidetes* and *Proteobacteria* in HSR supplementation groups of piglets indicated that HSR contributed to balancing the gut microbiota by facilitating beneficial bacterial colonization and inhibiting the proliferation of pathogenic bacteria. Ley et al. (2006b) reported the proportion of *Firmicutes* to *Bacteroides* was shown to increase in obese animals. In our study, supplementing HSR to diet increased the proportion of *Firmicutes* to *Bacteroides*, suggesting that HSR was conducive to the weight gain of piglets through altering microbiota constitution. Additionally, the accumulation of *Firmicutes* is described to connect with energy harvest and obesity (Turnbaugh et al., 2006), demonstrating that its accumulation might increase the calories acquisition from diet. Meanwhile, an increase in the abundance of *Tenericutes* was observed in piglets fed with Hu, which was similar with that of Liang et al. (2021), who reported that *Tenericutes* was increased in the intestinal tract of piglets fed *Clostridium butyricum*.

The dominant genera in the control groups were *Prevotella*, *Megasphaera,* and *Selenomonas* while those in treatment groups were *Megasphaera*, *Prevotella,* and *Lactobacillus*, suggesting that Hu administration contributed to modulating the intestinal flora of piglets. As expected, supplemental feeding with Hu with different form enhanced the abundance of *Lactobacillus* and HSJ group possessed the greatest abundance. Matsui et al. (2000) found that *Prevotella* as an active hemicellulose-decomposing bacterium exerted vital roles in degrading non-fibrous polysaccharides and proteins of plants. *Megaspaera* was a major inhabitant in pig intestine and responsible for transforming lactate into short chain fatty acids which are energy sources for the host (Zhang et al., 2018). Moreover, *Megaspaera* as a probiotic treatment was conducive to promoting intestinal health of pigs (Xie et al., 2017). It is reported that *Selenomonas* is a saccharolytic genera and positively related to the pre-weaned weight gain (Sawanon et al., 2011). Doron and Gorbach (2006) demonstrated that *Lactobacillus* was shown to reduce intestinal pH, prohibit various pathogenic bacteria proliferation, and balance the gut microbiota through producing lactic acid, propionic acid, and acetic acid. In the present study, HS groups presented the largest abundance of *Prevotellaceae* and *Ruminococcaceae* among the four groups. It is reported that *Prevotellaceae* and *Ruminococcaceae* are both negatively correlated with obesity (Zhang et al., 2015; Tun et al., 2017).

The microorganisms presented in the large intestine account for more metabolic activities (Zhao et al., 2015). In the present study, the abundances of amino acid transport and metabolism genes tended to increase in HSR supplementation groups. López-González et al. (2015) found that amino acid metabolism was described to provide the source of carbon and energy for microbiota. The cecum microbial functions in piglets supplemented with HS and HSJ were primarily reflected in replication, recombination and repair, defense mechanisms, and lipid transport and metabolism. Moreover, HS administration also possessed greater transcription and signal transduction mechanisms. Greater inorganic ion transport and metabolism were observed in HSR groups. The results of the study indicated that supplementation of Hu with three forms had diverse effects on the metabolic pathway of piglets, which may be attributed to the presence of different bioactive ingredients in Hu. Our results were consistent with a previous study by Jin et al. (2021), who illustrated that Xiao-Chai-Hu-Tang might exert an important role in colorectal cancer treatment by regulating different signaling pathways. Similarly, Xu et al. (2020) found that many pathways, such as PI3K–Akt signaling pathway, T cell receptor signaling pathway and calcium signaling pathway were shown to be potential signals of Huangqin-Baishao in conquering cancer.

In piglets, Hu supplementation enhances serum immunity, nutrient digestibility, and bacterial diversity, ultimately improving growth performance. Furthermore, piglets fed with HS exert more significant effects on nutrient digestibility, serum immunity, and growth performance. HSR supplemental diets contribute to increasing the abundance of beneficial bacteria in the cecum of piglets. Hu regulates these beneficial effects by mediating the diversity and abundance of gut microflora, optimizing bacterial structure, and facilitating beneficial bacterial colonization, thus improving bacterial diversity and intestinal health.

## Supplementary Material

txad139_suppl_Supplementary_Tables_S1

txad139_suppl_Supplementary_Tables_S2

txad139_suppl_Supplementary_Tables_S3

txad139_suppl_Supplementary_Tables_S4

## Data Availability

The 16S rRNA sequence data of samples have been submitted to the Sequence Read Archive (SRA BioProject no. PRJNA1029525) of NCBI for open access.
