# Supplementary material for: Dietary of different forms of Humulus scandens on growth performance and intestinal bacterial communities in piglets
Source: Transl Anim Sci. 2023 Dec 23;8:txad139. doi: 10.1093/tas/txad139 (PMC10782920; doi:10.1093/tas/txad139)
Supplement: txad139_suppl_Supplementary_Tables_S3 [file txad139_suppl_supplementary_tables_s3.doc]

**The title of the manuscript:** Dietary of different forms of *Humulus Scandens* on growth performance and intestinal bacterial communities in piglets

**The list of authors:** Lihong Hao, Cheng Wang, Huaizhong Wang, Meng Zhou, Yong Wang, Hongmei Hu*

**The journal name:** Translational Animal Science

**Supplementary Table S3.** Differences in gene function at the KEGG Level 1 of metabolic pathways in the cecum of piglets.

| **Characteristic** | **CG** | **HS** | **HSJ** | **HSR** | **SEM** | ***P*-value** |
| --- | --- | --- | --- | --- | --- | --- |
| Metabolism | 49.16 | 48.58 | 49.03 | 48.45 | 0.16 | 0.311 |
| Genetic information processing | 21.84^a^ | 21.32^ab^ | 21.71^a^ | 20.84^b^ | 0.11 | <0.001 |
| Environmental information processing | 10.29^b^ | 11.26^ab^ | 10.69^ab^ | 11.80^a^ | 0.19 | 0.021 |
| Cellular processes | 2.69 | 3.11 | 2.64 | 3.02 | 0.10 | 0.206 |
| Human diseases | 0.79 | 0.78 | 0.79 | 0.76 | 0.00 | 0.281 |
| Organismal systems | 0.81^a^ | 0.78^ab^ | 0.78^ab^ | 0.74^b^ | 0.01 | 0.011 |

Values are means of 6 replicates per treatment. ^a-b^Means with different superscripts in the same row within the trial differ (*P* < 0.05). CG, basal diet; HS, basal diet + Hu pulp; HSJ, basal diet + Hu juice; HSR, basal diet + Hu residue. SEM, standard error of the mean.

**Statistical analysis**

Data were statistically analyzed by the SPSS software (SPSS, Chicago, IL, USA) followed by One-way ANOVA analysis. For gut microbiota, pigs from each treatment served as the experimental unit, alongside a fixed effect of different forms of Hu in the statistical model. Duncan multiple-range tests defined the differences among treatments. The model utilized was as follows: Yijk = μ + Ti + eij, where Yijk = an observation, μ = the overall mean, Ti = effect of treatments, and eij = random error. All data were expressed as means with SEM. Statistical significance was defined *P* < 0.05.
