# Supplementary material for: Dietary of different forms of Humulus scandens on growth performance and intestinal bacterial communities in piglets
Source: Transl Anim Sci. 2023 Dec 23;8:txad139. doi: 10.1093/tas/txad139 (PMC10782920; doi:10.1093/tas/txad139)
Supplement: txad139_suppl_Supplementary_Tables_S4 [file txad139_suppl_supplementary_tables_s4.doc]

**The title of the manuscript:** Dietary of different forms of *Humulus Scandens* on growth performance and intestinal bacterial communities in piglets

**The list of authors:** Lihong Hao, Cheng Wang, Huaizhong Wang, Meng Zhou, Yong Wang, Hongmei Hu*

**The journal name:** Translational Animal Science

**Supplementary Table S4.** Differences in gene function at the KEGG Level 2 of ortholog functional predictions in the cecum of piglets.

Values are means of 6 replicates per treatment. ^a-c^Means with different superscripts in the same row within the trial differ (*P* < 0.05). CG, basal diet; HS, basal diet + Hu pulp; HSJ, basal diet + Hu juice; HSR, basal diet + Hu residue. SEM, standard error of the mean.

| **Characteristic** | **CG** | **HS** | **HSJ** | **HSR** | **SEM** | ***P*-value** |
| --- | --- | --- | --- | --- | --- | --- |
| Translation, ribosomal structure and biogenesis | 9.82^ab^ | 9.95^ab^ | 10.04^a^ | 9.75^b^ | 0.04 | 0.014 |
| Transcription | 6.13^b^ | 6.87^a^ | 6.43^b^ | 6.49^ab^ | 0.07 | <0.001 |
| Replication, recombination and repair | 6.32^b^ | 6.55^a^ | 6.51^a^ | 6.26^b^ | 0.03 | <0.001 |
| Cell cycle control and cell division | 1.58 | 1.49 | 1.52 | 1.54 | 0.01 | 0.088 |
| Defense mechanisms | 1.96^c^ | 2.36^a^ | 2.15^b^ | 1.90^c^ | 0.04 | <0.001 |
| Signal transduction mechanisms | 3.03^b^ | 3.37^a^ | 3.09^b^ | 3.13^ab^ | 0.04 | 0.006 |
| Cell wall, membrane and envelope biogenesis | 7.43^a^ | 7.38^a^ | 7.53^a^ | 6.92^b^ | 0.06 | <0.001 |
| Cell motility | 1.13 | 0.93 | 0.83 | 1.09 | 0.05 | 0.054 |
| Intracellular trafficking and secretion | 1.58 | 1.44 | 1.48 | 1.55 | 0.01 | 0.051 |
| Posttranslational modification and protein turnover | 3.83^a^ | 3.73^ab^ | 3.83^a^ | 3.63^b^ | 0.03 | 0.005 |
| Energy production and conversion | 6.07 | 5.99 | 5.97 | 6.04 | 0.02 | 0.495 |
| Carbohydrate transport and metabolism | 6.97 | 6.80 | 6.87 | 7.05 | 0.04 | 0.118 |
| Amino acid transport and metabolism | 10.20^a^ | 9.73^b^ | 9.90^b^ | 10.46^a^ | 0.07 | <0.001 |
| Nucleotide transport and metabolism | 4.13^ab^ | 4.15^ab^ | 4.27^a^ | 4.08^b^ | 0.02 | 0.017 |
| Coenzyme transport and metabolism | 5.13^a^ | 4.41^c^ | 4.76^b^ | 5.04^a^ | 0.07 | <0.001 |
| Lipid transport and metabolism | 2.39^b^ | 2.61^a^ | 2.53^a^ | 2.28^c^ | 0.03 | <0.001 |
| Inorganic ion transport and metabolism | 5.47^b^ | 5.56^ab^ | 5.49^b^ | 5.67^a^ | 0.02 | <0.001 |
| Secondary metabolites biosynthesis and transport | 0.61 | 0.54 | 0.58 | 0.57 | 0.01 | 0.076 |

**Statistical analysis**

Data were statistically analyzed by the SPSS software (SPSS, Chicago, IL, USA) followed by One-way ANOVA analysis. For gut microbiota, pigs from each treatment served as the experimental unit, alongside a fixed effect of different forms of Hu in the statistical model. Duncan multiple-range tests defined the differences among treatments. The model utilized was as follows: Yijk = μ + Ti + eij, where Yijk = an observation, μ = the overall mean, Ti = effect of treatments, and eij = random error. All data were expressed as means with SEM. Statistical significance was defined *P* < 0.05.
